# Fibroblast Growth Factor 1 Promotes Rat Stem Leydig Cell Development

**DOI:** 10.3389/fendo.2019.00118

**Published:** 2019-03-08

**Authors:** Lanlan Chen, Xiaoheng Li, Yiyan Wang, Tiantian Song, Huitao Li, Lubin Xie, Linchao Li, Xianwu Chen, Leikai Ma, Yong Chen, Yao Lv, Xingwang Li, Ren-Shan Ge

**Affiliations:** ^1^Department of Anesthesiology, The Second Affiliated Hospital and Yuying Children's Hospital of Wenzhou Medical University, Wenzhou, China; ^2^Department of Anesthesiology, Taizhou People's Hospital, The Fifth Hospital Affiliated Nantong University, Taizhou, China; ^3^Department of Obstetrics and Gynecology, The Second Affiliated Hospital and Yuying Children's Hospital of Wenzhou Medical University, Wenzhou, China

**Keywords:** fibroblast growth factor 1, stem leydig cell, differentiation, proliferation, testosterone

## Abstract

Fibroblast growth factor 1 (FGF1) is reported to be expressed in the testis. How FGF1 affects stem Leydig cell development remains unclear. Here, we report the effects of FGF1 on rat stem Leydig cell development in an ethane dimethane sulfonate (EDS)-treated model. FGF1 (100 ng/testis) significantly increased serum testosterone level, increased PCNA-positive Leydig cell percentage and Leydig cell number, but down-regulated the expression of *Lhcgr, Star, Cyp11a1, Hsd3b1, Cyp17a1*, and *Hsd11b1* in Leydig cells *per se*, after its daily intratesticular injection from post-EDS day 14 for 14 days. Primary culture of the seminiferous tubules showed that FGF1 stimulated EdU incorporation to stem Leydig cells but blocked the differentiation into the Leydig cell lineage, possibly via FGFR1-mediated mechanism. In conclusion, FGF1 promotes stem Leydig cell proliferation but blocks its differentiation.

## Introduction

The adult Leydig cell (ALC) is a cell type in the mammalian testis that secretes androgen. ALCs derive from differentiation of stem Leydig cells (SLCs) during the pubertal period. Once established, they turn over slowly at adulthood. In several mammalian species, including rats, however, when males are administered with ethane dimethane sulfonate (EDS), a chemical that only kills ALCs in the testis, a new population of ALCs develops (1, 2). These newly-formed ALCs arise from the undifferentiated SLCs (3). Four days after one-dose administration of EDS to the rat, ALCs undergo apoptosis to disappear and then SLCs begin to proliferate (1, 2). On day 21 after EDS dosing, progenitor cells develop as shown by the expression of the Leydig cell lineage biomarkers: lutropin-choriogonadotropic hormone receptor (LHCGR), high-density lipoprotein receptor (SCARB1), steroidogenic acute regulatory protein (STAR), cholesterol side-chain-cleaving enzyme (CYP11A1), 3β- 3β-hydroxysteroid dehydrogenase/Δ(5)-Δ(4)isomerase type I (HSD3B1), cytochrome 17α-hydroxylase/17,20 lyase/17,20 desmolase (CYP17A1), and steroid 5α-reductase 1 (SRD5A1) (4, 5). Thereafter, these progenitor cells proceed into immature-type cells as judged by the appearance of the biomarkers, 11β-hydroxysteroid dehydrogenase 1 (HSD11B1) and 17β-hydroxysteroid dehydrogenase 3 (HSD17B3) until 28 days after EDS. Immature cells mature into ALCs with silencing of *Srd5a1* gene until 56 days after EDS (4, 5).

Previous studies indicate that SLCs stay outside the seminiferous tubules (STs) of rat testis (6, 7) and they are capable of differentiating into ALCs under the induction of a Leydig cell differentiation medium (LCDM), which contains insulin-transferrin-selenium, LH, and lithium ion (7). In the process from SLCs into the adult-type form, plenty of endocrine and paracrine factors control the proliferation and differentiation of these SLCs (8). One set of growth factors are fibroblast growth factors (FGFs). FGF family is composed of 23 polypeptides that possess multiple biological functions, including development, cell growth, metabolic regulation, as well as wound healing and repair (9). The first member in the FGF family is fibroblast growth factor 1 (FGF1), which has been reported to be present in liver, kidney, brain, and testis (10). FGF1 is capable of binding and activating all FGF receptors (FGFRs) (11). FGFRs are kinase receptors that are composed of the four classic FGFR isotypes 1-4 (FGFR1-4) and are tyrosine kinases, which mediate FGF1 actions at the cellular level (12). FGF1 requires the presence of heparan sulfate proteoglycans (HSPG) to exert its effects, (13) mainly acting as autocrine or paracrine factor (14). FGF1 is immunologically identified in Leydig, Sertoli, and germ cells in rat testis (15). Previous studies showed that FGF1 was able to inhibit LH-stimulated regulation of HSD3B1 and SRD5A1 and to lower LHCGR number in rat immature Leydig cells, (16) but it stimulated basal androgen production in these cells (13). Here, we interrogate FGF1 roles on SLC development and dissect the underlying mechanism.

## Materials and Methods

### Chemicals and Kits

Recombinant FGF1 peptide was obtained from PeproTech (Rocky Hill, NJ). Immulite2000 Total Testosterone kit is a commercial product of Sinopharm Group Medical Supply Chain Services (Hangzhou, China). Heparin (HA) and PD173074 (PD) were purchased from Sigma (St. Louis, MO). Culture media and Click-iT EdU Imaging Kit were obtained from Invitrogen (Carlsbad, CA). EDS is a commercial product of Pterosaur Biotech (Hangzhou, China).

### Reanalysis of Microarray Data of Cells in the Leydig Cell Lineage

Transcriptome data of stem, progenitor, immature, and adult Leydig cells were deposited in the NIH Omnibus (accession #: GSE26703) and the analysis was published in our previous study (17). In this study, we performed reanalysis of the data for the expression of FGF receptors.

### EDS-treated Leydig Cell Regeneration in Rats

Eighteen male Sprague Dawley rats (age 60 days old) were acquired from Shanghai Laboratory Animal Center (Shanghai, China). They were shipped to Wenzhou Medical University and adjusted for a week in the new environment. To ablate ALCs from the testis, each rat was administered with EDS (75 mg/kg body weight) via intraperitoneal way. The solvent of EDS is dimethyl sulfoxide: water (1: 3, v/v). Male rats were randomly divided into 3 groups and each group had 6 rats. We intratesticular injected 0 (normal saline), 10, or 100 ng/testis FGF1 from day 14 to day 28 after EDS administration. The intratesticular injection volume was 20 μl and the preliminary data showed that this volume did not influence the testis. In order to exclude the systemic effects due to the hypothalamus-pituitary-testis axis function, we selected intratesticular injection regimen. Fourteen days after FGF1 treatment, rats were euthanized by CO_2_ and blood samples were collected for sera. We measured testosterone, LH, and follicle-stimulating hormone (FSH) levels in these serum samples. We collected total RNAs from the testis to perform real-time quantitative PCR (qPCR) and proteins to perform Western blotting analysis. The contralateral testis was immerged in a bottle containing Bouin's solution and was used for immunohistochemical staining. The animal protocol was approved by Animal Care and Use Committee of Wenzhou Medical University.

### Serum and Medium Testosterone Measurement

Testosterone concentrations were detected by Immulite2000 Total Testosterone kit. Normal male rat serum serves as the internal quality control. The minimum detection concentration of testosterone is 0.2 ng/ml.

### ELISA for Serum LH and FSH Levels

LH and FSH levels were assayed using ELISA kits as described previously (18). Serum sample and assay solution were mixed and incubated for 2 h at room temperature. Peroxidase-conjugated IgG anti-LH or anti-FSH liquid was then mixed and incubated for 2 h at room temperature. The substrate buffer was followed and the plate was stored in the dark place for 30 min. The reaction stop solution was added to end the enzyme reaction. The parameters of microplate reader were setup as 550 nm with correction wavelength at 450 nm.

### Quantitative Real-Time PCR (qPCR)

We picked a piece of testis and put it in the Trizol solution (Invitrogen, Carlsbad, CA) and extracted the total RNAs. After the RNAs were purified, a NanoDrop 2000 was used to read RNA concentrations. CDNAs of these RNA samples were reverse-transcribed according to a previously described method (19). We used SYBR Green qPCR Kit (Roche, Basel, Switzerland) to measure the following Leydig cell mRNAs: *Lhcgr, Scarb1, Star, Cyp11a1, Hsd3b1, Cyp17a1, Hsd17b3, Srd5a1*, and *Hsd11b1*. 7.5 μl SYBR Green Mix, 0.75 μl forward primer/0.75 μl reverse primer, 0.02 μg diluted cDNA and 4 μl RNA-free water and samples were mixed. The qPCR program includes 95°C for 5 min, 40 cycles of 95°C for 10 s, and 60°C for 30 s. Melting curve analysis and gel electrophoresis were selected to identify the specificity. A standard curve using Ct values was generated to calculate the concentrations of target mRNA. The target mRNA levels were adjusted to *Rps16*. *Rps16* serves the internal control. Supplementary Table S1 includes the primer information.

### Western Blot Analysis

Western blotting technique was selected as previously described (20). Briefly, we cut a piece of the testis or ST and dipped it into RIPA lysis buffer (Bocai Biotechnology, China) and homogenized it in a glass homogenizer. BCA Protein Assay Kit (Takara, Japan) was selected to measure protein concentrations. We loaded 30 μg of protein per sample into the gel and separated the proteins using denature electrophoresis method. When the electrophoresis was ended, the proteins were transferred onto the nitrocellulose membrane. Five percent Non-fat milk was used to block non-specific binding and the following primary antibodies [LHCGR, SCARB1, HSD3B1, CYP17A1, HSD17B3, HSD11B1, phosphorylated AKT1 (pAKT1), AKT1, phosphorylated AKT2 (pAKT2), AKT2, phosphorylated EKR1/2 (pERK1/2), and ERK1/2] were bound to the membrane individually. Supplementary Table S2 has the antibody information. After cleaning with several washes, the membrane was incubated with HRP-conjugated anti-rabbit (1:2,000, Bioword, USA) for 2 h. Super-Signal West Pico chemiluminescent substrate (Pierce Biotechnology, Radford, IL) was added and the band photos were taken in Universal Hood II (Bio-Rad, Hercules, CA). ACTB is the protein control. The band intensities were read by Bio-Rad Image Lab (Hercules, CA). The data of target proteins were normalized to ACTB.

### Preparation of Testis Tissue Array

We prepared a tissue array as previously described (21). We selected one testis per rat (6 rats per group), cut in 8 discs, of which two pieces were randomly selected. We further cut these two discs into 4 pieces each and one piece was randomly selected from these 8 pieces. We embedded the testis piece in paraffin in a tissue array container. We cut tissue array block into 6 μm-thick sections. From these sections, 10 slides were randomly selected for the following immunohistochemical staining and the other 10 slides were selected for immunofluorescent staining.

### Immunohistochemical and Immunofluorescent Staining of the Testis

Immunohistochemical staining kit from Vector Laboratories (Burlingame, CA) was used according to the manufacturer's instruction. The tissue selections were selected for antigen retrieval in 10 mM (pH 6.0) of citrate buffer in a microwave machine. Slides were immerged in 0.5% of H_2_O_2_ in methanol for 30 min to remove endogenous peroxidase activity. Two antibodies, CYP11A1 (biomarker of Leydig cells) or HSD11B1 (biomarkers for Leydig cells at the advanced stage) polyclonal antibodies, were used. Antibodies were diluted 1:200. Diaminobenzidine solution was added to show the brown color of the target protein. Counterstaining is Mayer hematoxylin. Non-immune rabbit IgG was used as the negative control.

We performed immunofluorescent staining of PCNA and CYP11A1 to identify the proliferative Leydig cells. Alexa-conjugated anti-rabbit or anti-mouse IgG second antibodies (1:500) were selected. Counterstaining is DAPI solution. Olympus fluorescent microscope (Olympus, Japan) was used to identify the positive cells. The CYP11A1 (green color in the cytoplasm) represents the Leydig cell and PCNA (red color in the nucleus) represents the proliferating cell.

### Counting Leydig Cells

To count CYP11A1-positive or HSD11B1-positive Leydig cells, a fractionator technique was used for above tissue-array sections as previously described (22). In brief, under the live image of a digital camera with a 10 × objective and fixed point of the “upper” sections, we counted cells of total microscopic field. We calculated the total number of Leydig cells by multiplying Leydig cell number counted in a known fraction of the testis by the inverse of the sampling probability.

### Culturing SLCs on the Surface of STs

One rat was treated with EDS as above and testes were collected 7 days after administration of EDS (1, 23). STs were separated from the interstitium as previously described (6, 24). The STs were distributed randomly into a 12-well plate, with each well-containing equivalent amount of ST fragments of total length (about 1 inch). STs were cultured in LCDM, which is composed of DMEM/F12 medium (pH 7.2), supplemented with 5 mM insulin-transferrin-selenium, 5 ng/ml LH, and 5 mM lithium ion, 0.1% bovine serum albumin (BSA), in a humidified atmosphere of 5% CO_2_ at 37°C in a 12-well culture plate (Corning, NY) for up to 2 weeks. Various concentrations of FGF1 and/or FGFR1 inhibitor PD173074 (PD, 1 μM), and/or HSPG antagonist heparin (HA, 1 μg/ml) were co-incubated in LCDM to study the effects of FGF1 on SLC differentiation. For indirect assay of SLC proliferation, various concentrations of FGF1 and/or FGFR1 inhibitor PD173074 (PD, 1 μM), and/or HSPG antagonist heparin (HA, 1 μg/ml) were incubated with the STs in M199 media for 7 days and then the tubules were switched in LCDM and cultured for additional 2 weeks. At the end of treatment, ALCs were induced from SLCs, secreting testosterone into the medium. The medium samples were collected and testosterone levels were assayed as above.

### EdU Incorporation Into SLCs

EdU incorporation into SLCs was measured by EdU (EdU) Alaxa Fluor Kit (Life Technologies, U.S.) as previously described (25). In brief, the freshly isolated STs were cultured in M199 medium and treated with 0–100 ng/ml FGF1 for 7 days. EdU (1:1,000 dilution) was added to the STs and incubated for additional 24 h. STs were washed with PBS containing 3% BSA. The STs were fixed in 4% paraformaldehyde for 30 min. STs were washed and stained. Olympus fluorescence microscope (Olympus, Japan) was used to capture images. EdU-positive cells (green color in the nucleus) were counted under using the ImageProPlus 7.0 software (Media Cybernetics, Rockville, MD).

### Statistical Analysis

Mean ± SEM was used to present the data. *P* < 0.05 was considered to be statistically significant. One-way ANOVA and then *ad hoc* Dunnett's multiple comparison test or paired student *t*-test after Sidak adjustment (for Western blotting analysis only) were used in the SigmaStat software (Systat Software, Richmond, CA) to compare them with the control.

## Results

### Leydig Cells Express Fgfr1 and Fgfr3

We reanalyzed the microarray data of stem, progenitor, immature, and adult Leydig cells in the Leydig cell lineage for the *Fgfr* expression. *Fgfr1* and *Fgfr3* were expressed in the Leydig cell lineage with *Fgfr1* levels being higher than those of *Fgfr3* in the Leydig cell lineage (Figure S1). This indicates that FGF1 can act primarily via FGFR1.

### FGF1 Stimulates Testosterone Synthesis *in vivo*

**Figure 1:**
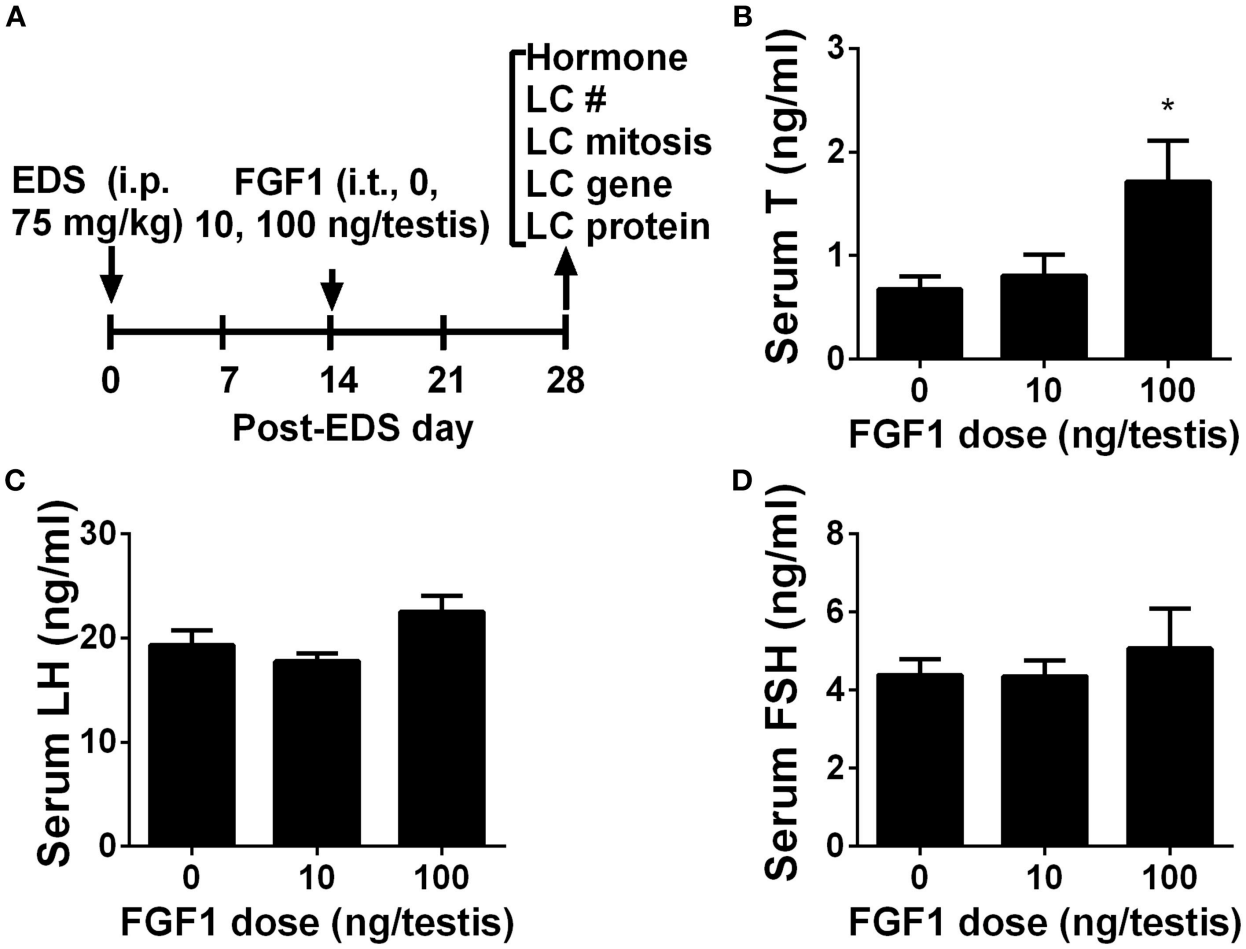
Serum testosterone, LH, and FSH levels after *in vivo* FGF1 treatment **(A)** Scheme of the animal experiment of intratesticularly injecting (i.t.) 0, 10, and 100 ng/testis FGF1 on post-EDS day 14 for 14 days; EDS, ethane dimethane sulfonate; LC, Leydig cell; i.p., intraperitoneal injection; **(B)** Serum testosterone (T) levels; **(C)** LH levels; **(D)** FSH levels. Mean ± SEM, *n* = 6. Asterisk (^*^) designates significant difference at *P* < 0.05 when compared to the control (0 ng/testis FGF1).

We interrogated whether FGF1 affects rat Leydig cell regeneration. The EDS model mimics Leydig cell development (4, 26, 27). We administered EDS to the rat to ablate all ALCs and then administered FGF1 (0, 10, or 100 ng/testis/day) to rats via intratesticular injection for 14 days (Figure 1A). At the end of the treatment, FGF1 did not influence the body and testis and relative testis weights (divided by body weights) after compared to the control values (Supplementary Table S3). FGF1 significantly stimulates testosterone production at a dose of 100 ng/testis (Figure 1B). However, FGF1 did not change serum LH (Figure 1C) and FSH (Figure 1D) levels. These data indicate that the action of FGF1 was within the testis.

### FGF1 Increases Leydig Cell Number *in vivo*

**Figure 2:**
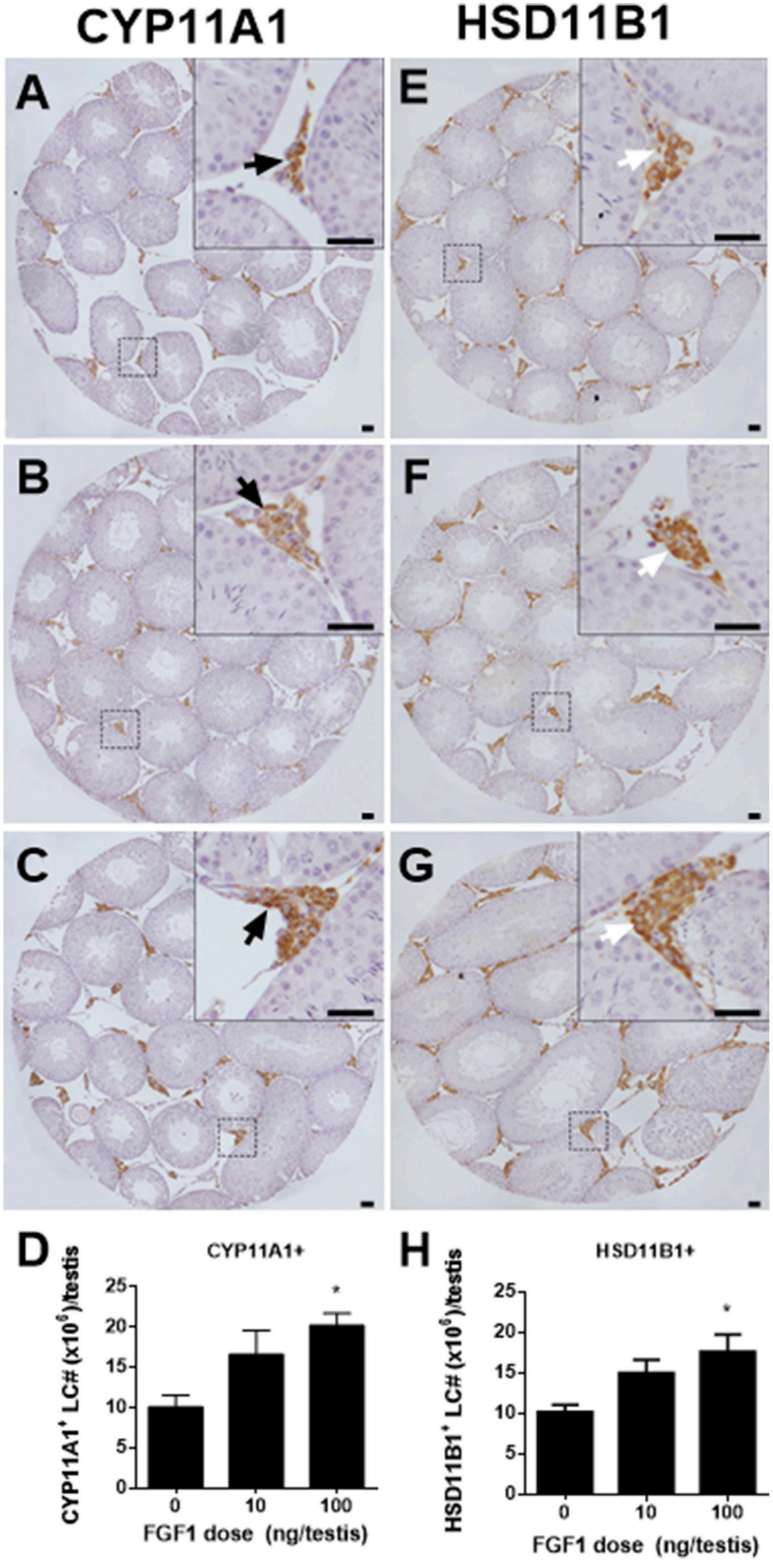
Morphology and cell number of Leydig cells after *in vivo* FGF1 treatment. Immunohistochemical staining of CYP11A1 **(A–C)** and HSD11B1 **(E–G)** of the testes from the rats treated with 0, 10, and 100 ng/testis FGF1 on post-EDS day 14 for 14 days. **(A,E)** the control (0 ng/testis FGF1); **(B,F)** 10 ng/testis FGF1; **(C,G)**: 100 ng/testis FGF1; **(D,H)** quantitative data. Black arrow designates CYP11A1 positive Leydig cells. White arrow designates HSD11B1 positive Leydig cells. Bar = 50 μm. Mean ± SEM, *n* = 6. Asterisk (^*^) designates significant difference at *P* < 0.05 when compared to the control (0 ng/testis FGF1).

We counted CYP11A1-positive and HSD11B1-positive Leydig cells in the EDS-administered testis and found that FGF1 dose-dependently increased the number of CYP11A1-positive and HSD11B1-positive Leydig cells at doses of 10 and 100 ng/testis with statistical significance being recorded in 100 ng/testis group (Figures 2A–H), indicating that FGF1 stimulates the proliferation of SLCs and progenitor cells.

### FGF1 Stimulates Leydig Cell Mitosis *in vivo*

**Figure 3:**
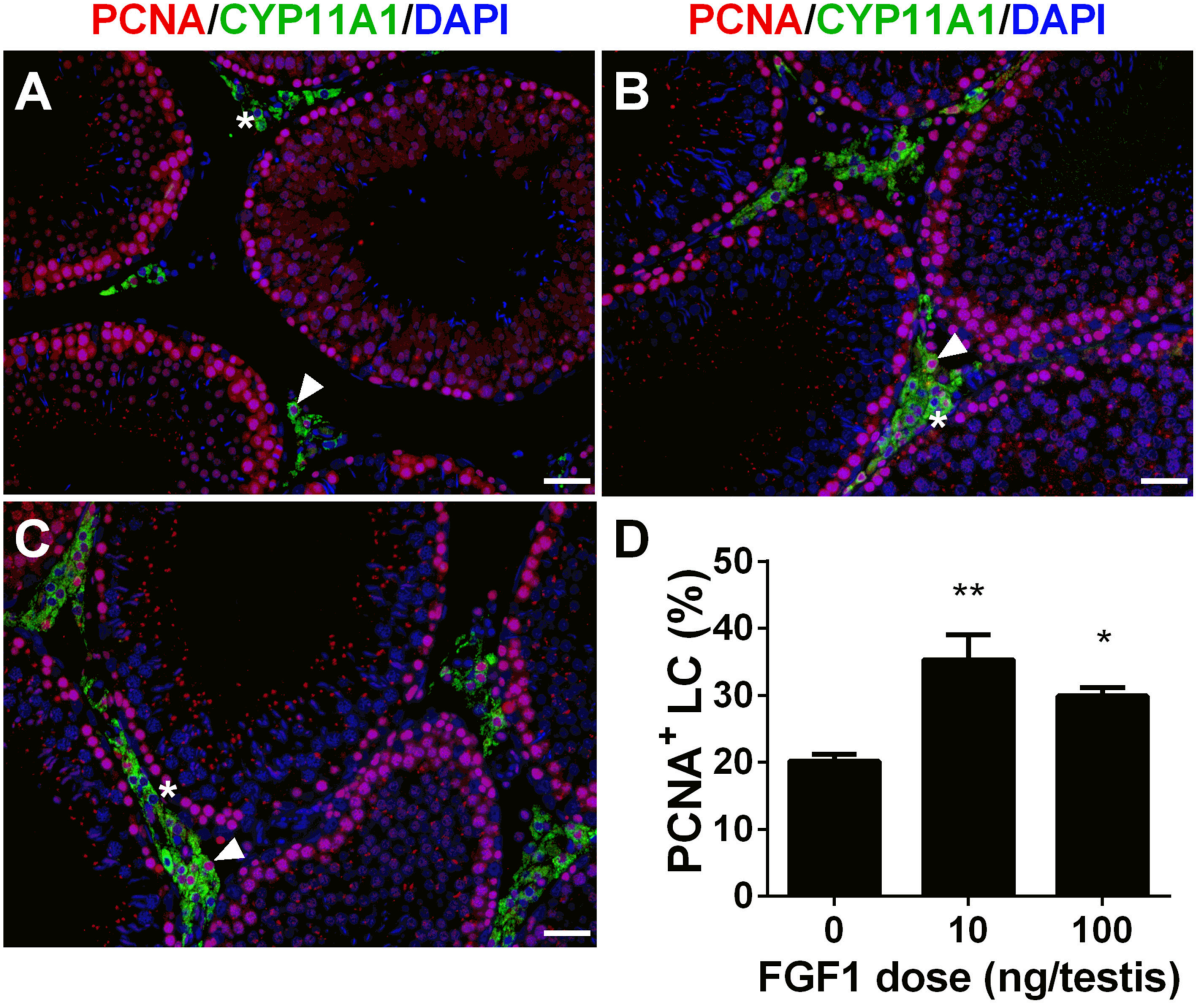
FGF1 promotes proliferation of Leydig cells *in vivo*. Immunofluorescent staining of PCNA (red color in the nucleus) and CYP11A1 (green color in the cytoplasm) of cells in the testes from the rats treated with 0 **(A)**, 10 **(B)**, and 100 ng/testis FGF1 **(C)** on post-EDS day 14 for 14 days; **(D)**: quantitative data of percentage of PCNA-positive and CYP11A1 positive cells. White arrow designates PCNA-positive Leydig cells. White “^*^” designates PCNA-negative Leydig cells. Bar = 50 μm. Mean ± SEM, *n* = 6. Asterisk (^*^, ^**^) designate significant difference at *P* < 0.05 and 0.01, respectively, when compared to the control (0 ng/testis FGF1).

We calculated PCNA-positive and CYP11A1-positive cells. As shown in Figure 3, double stainings of PCNA and CYP11A1 demonstrated that FGF1 increased the Leydig cell proliferative rate. This result indicates that FGF1 stimulates Leydig cell mitosis.

### FGF1 Controls Leydig Cell Specific Gene Expression *in vivo*

**Figure 4:**
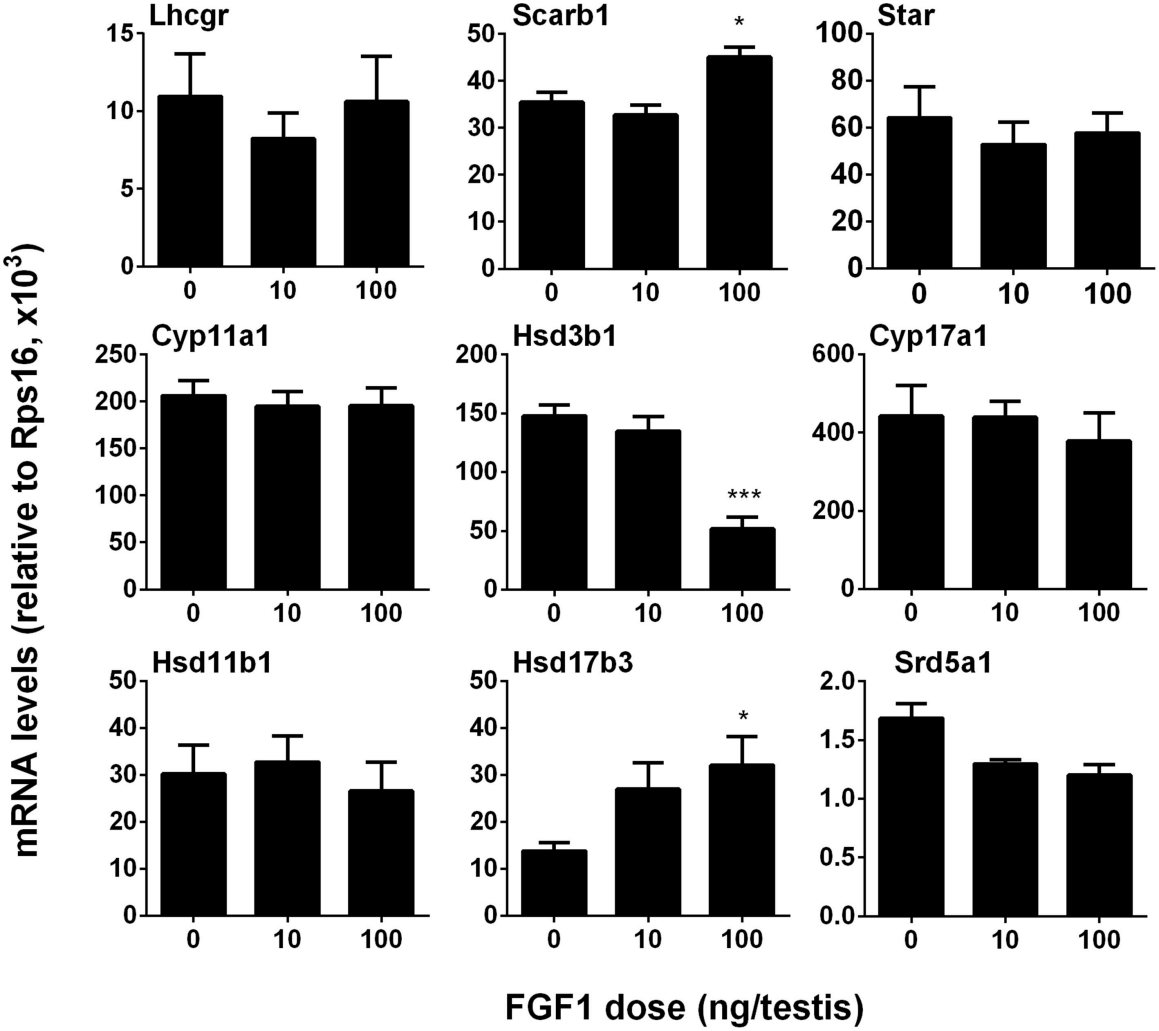
FGF1 affects the expression of Leydig cell-specific genes *in vivo*. The mRNA levels of *Lhcgr, Scarb1, Star, Cyp11a1, Hsd3b1, Cyp17a1, Hsd17b3, Srd5a1*, and *Hsd11b1* were analyzed by qPCR in the testes from rats treated with 0, 10, and 100 ng/testis FGF1 on post-EDS day 14 for 14 days. Mean ± SEM, *n* = 6, Asterisk (^*^, ^***^) designate significant difference at *P* < 0.05 and 0.001, respectively, when compared to the control (0 ng/testis FGF1).

We measured the mRNA levels of ALC-specific genes. The results showed that FGF1 significantly increased *Scarb1* and *Hsd17b3* mRNA levels and down-regulated *Hsd3b1* expression at a dose of 100 ng/testis when compared to the control (Figure 4). This indicates that FGF1 increases testosterone levels at 100 ng/testis via increasing the expression of *Scarb1* and *Hsd17b3*. Interestingly, when normalized to CYP11A1-positive ALCs, the expression of all genes, including *Lhcgr, Scarb1, Star, Cyp11a1, Hsd3b1, Cyp17a1, Hsd11b1*, and *Srd5a1*, except *Hsd17b3*, were down-regulated by 10 and 100 ng/testis FGF1 (Figure S2), indicating that FGF1 down-regulates the expression of most Leydig cell genes per se. This further suggests that serum testosterone level after FGF1 treatment relies on the balance of Leydig cell number and Leydig cell-specific mRNA levels.

### FGF1 Controls Leydig Cell Specific Protein Levels *in vivo*

**Figure 5:**
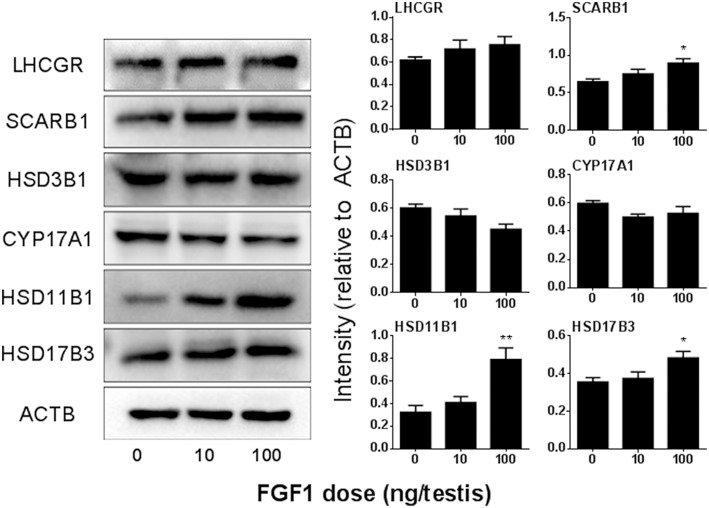
FGF1 affects Leydig cell-specific protein levels *in vivo*. **Left**: gel; **Right**: quantitative data of each protein. The protein levels of Lhcgr, SCARB1, HSD3B1, Cyp17a1, HSD11B1, HSD17B3, and ACTB (control) were analyzed by Western blot in the testes from rats treated with 0, 10, and 100 ng/testis FGF1 on post-EDS day 14 for 14 days. Mean ± SEM, *n* = 3–5. Asterisk (^*^) designates significant difference at *P* < 0.05 when compared to the control (0 ng/testis FGF1).

We detected the protein levels of ALCs after Western blotting the intensity analysis and demonstrated that the protein levels change according to their respective mRNA levels (Figure 5).

### FGF1 Regulates AKT1, AKT2, and ERK1/2 Pathways *in vivo*

**Figure 6:**
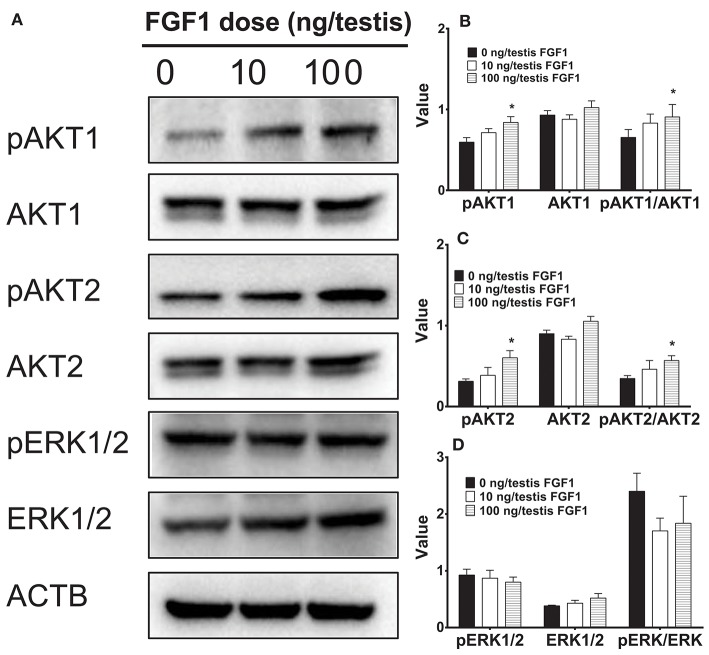
The kinase and phosphorylated kinase protein levels of the testis of rats with or without FGF1 treatment *in vivo*. **(A)** gel; **(B-D)** quantitative data. The protein levels of pAKT1, AKT1, pAKT2, AKT2, pERK1/2, ERK1/2, and ACTB (control) were analyzed by Western blot in the testes from rats treated with 0, 10, and 100 ng/testis FGF1 on post-EDS day 14 for 14 days. Values include the ACTB-adjusted levels of pAKT1, AKT1, pAKT2, AKT2, pERK1/2, and ERK1/2 as well as the ratios of pAKT1/AKT1, pAKT2/AKT2, and pERK/ERK. Mean ± SEM, *n* = 4–5. Asterisk (^*^) designates significant difference at *P* < 0.05 when compared to the control (0 ng/testis FGF1).

Several studies have suggested that AKT1, AKT2, and ERK1/2 pathways might control Leydig cell function (28–30). Here, we interrogated the downstream signals of FGF1 in the testis. FGF1 significantly elevated pAKT1 and pAKT2 levels and pAKT1/AKT1 and pAKT2/AKT2 ratio at 100 ng/testis. However, FGF1 did not alter phospho-ERK1/2 (pERK1/2) and EKR1/2 levels and its ratio (Figure 6). This indicates that FGF1 acts via AKT1/AKT2 pathways.

### FGF1 Blocks SLC Differentiation *in vitro*

**Figure 7:**
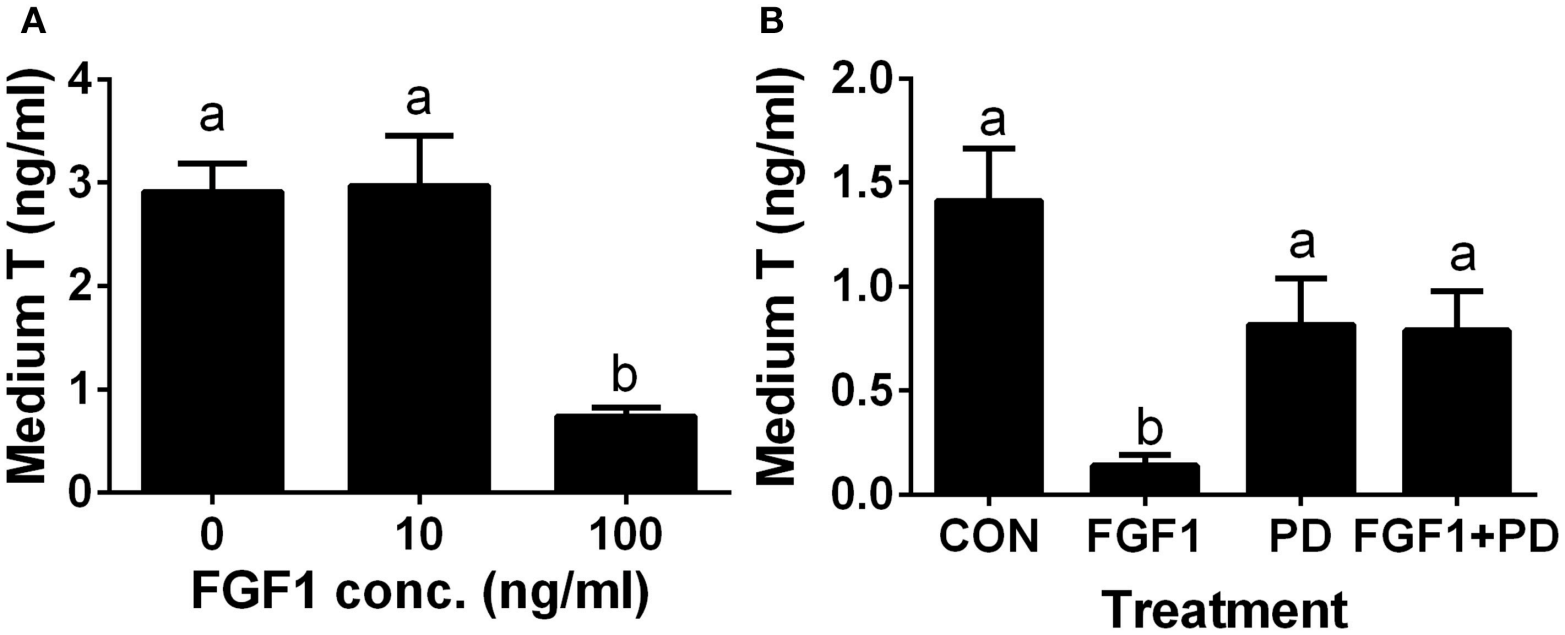
FGF1 affects medium testosterone levels *in vitro*. EDS-treated seminiferous tubules were cultured in LCDM for 14 days. **(A)** the effects of different concentrations of FGF1 on medium testosterone (T) levels; **(B)**, medium testosterone (T) levels after control (CON, no agent), 10 ng/ml FGF1 (FGF1), 1 μM PD173074 (a FGFR1 antagonist, PD), and 10 ng/ml FGF1 + 1 μM PD (FGF1 + PD) for 14 days. Mean ± SEM, *n* = 12. Identical letters designate no significant differences at *P* < 0.05 between two groups.

Since the ALC regeneration processes in a period of 28 days after administration of EDS treatment covers the differentiation of SLCs into progenitor cells and further into immature Leydig cells, (4). we interrogated whether FGF1 controls the differentiation of SLCs into the ALC lineage. We used an *in vitro* SLC differentiation model (7). SLCs can be induced to differentiate into ALCs in LCDM, which secrete testosterone (7). Indeed, FGF1 significantly lowered testosterone levels at 100 ng/ml (Figure 7A). Then, we used a potent FGFR1 receptor antagonist, PD173074, to dissect the mechanism of FGF1. Indeed, FGF1 (100 ng/ml) significantly inhibited testosterone production and PD173074 (1 μM) alone had no effect on testosterone levels (Figure 7B). However, PD173074 completely reversed FGF1-mediated inhibition on testosterone levels (Figure 7B). These results suggest that FGF1 blocks SLC commitment into ALC lineage via acting on FGFR1.

#### FGF1 Down-Regulates Leydig Cell Gene Expression *in vitro*

**Figure 8:**
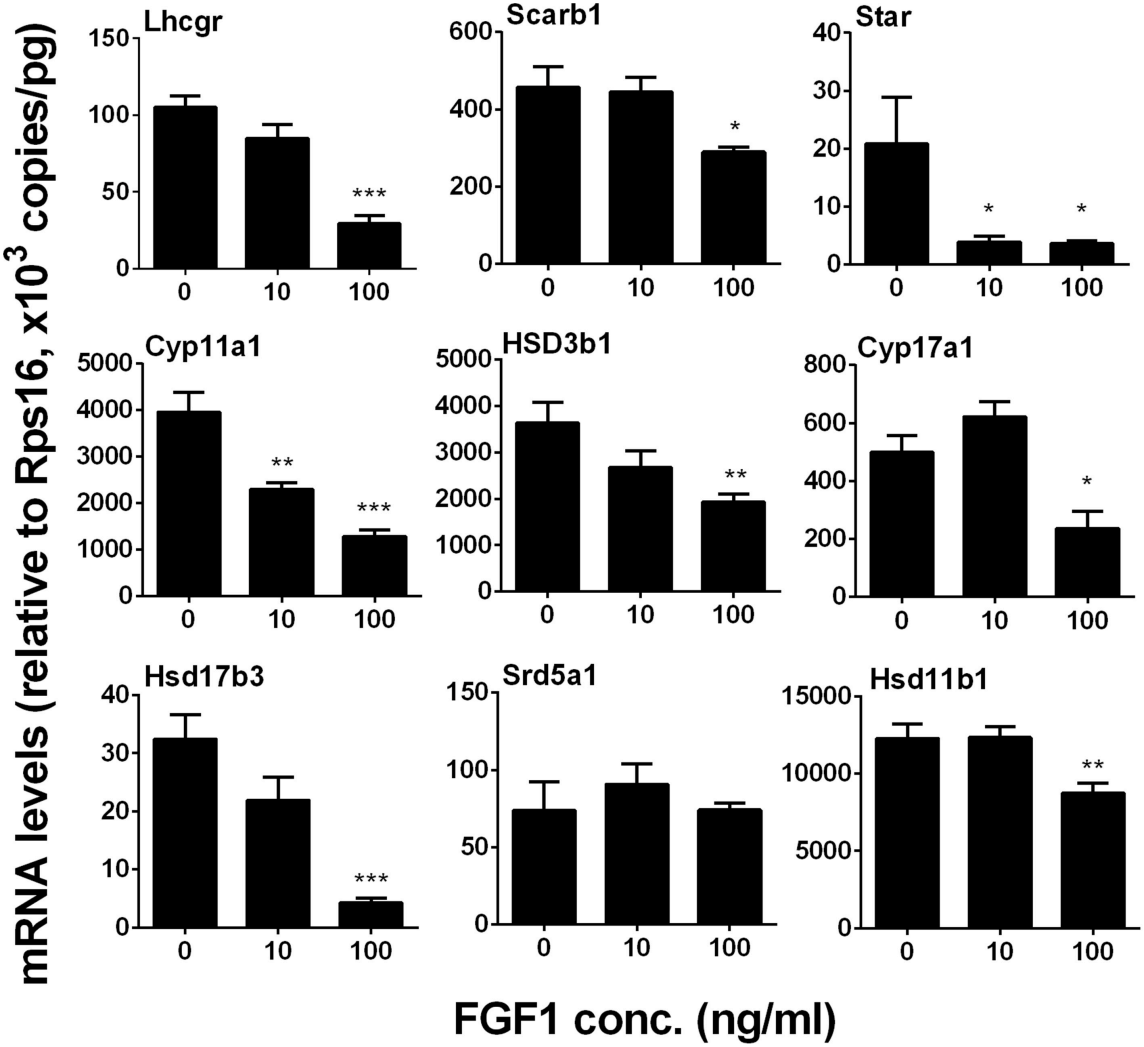
FGF1 affects the expression of Leydig cell-specific genes *in vitro*. The mRNA levels of *Lhcgr, Scarb1, Star, Cyp11a1, Hsd3b1, Cyp17a1, Hsd17b3, Srd5a1*, and *Hsd11b1* were analyzed by qPCR in the seminiferous tubules treated with 0, 10, and 100 ng/ml FGF1 in LCDM for 14 days. Mean ± SEM, *n* = 6, Asterisks (^*^, ^**^, ^***^) designate significant differences at *P* < 0.05, 0.01, and 0.001, respectively, when compared to the control (0 ng/ml FGF1).

We measured *Lhcgr, Scarb1, Star, Cyp11a1, Hsd3b1, Cyp17a1, Hsd17b3, Srd5a1*, and *Hsd11b1* levels. We identified that FGF1 at 10 or 100 ng/ml decreased *Lhcgr, Scarb1, Star, Cyp11a1, Hsd3b1, Cyp17a1, Hsd17b3*, and *Hsd11b1* 1evels (Figure 8). This indicates that FGF1 blocks SLC differentiation.

### FGF1 Stimulates SLC Proliferation *in vitro*

**Figure 9:**
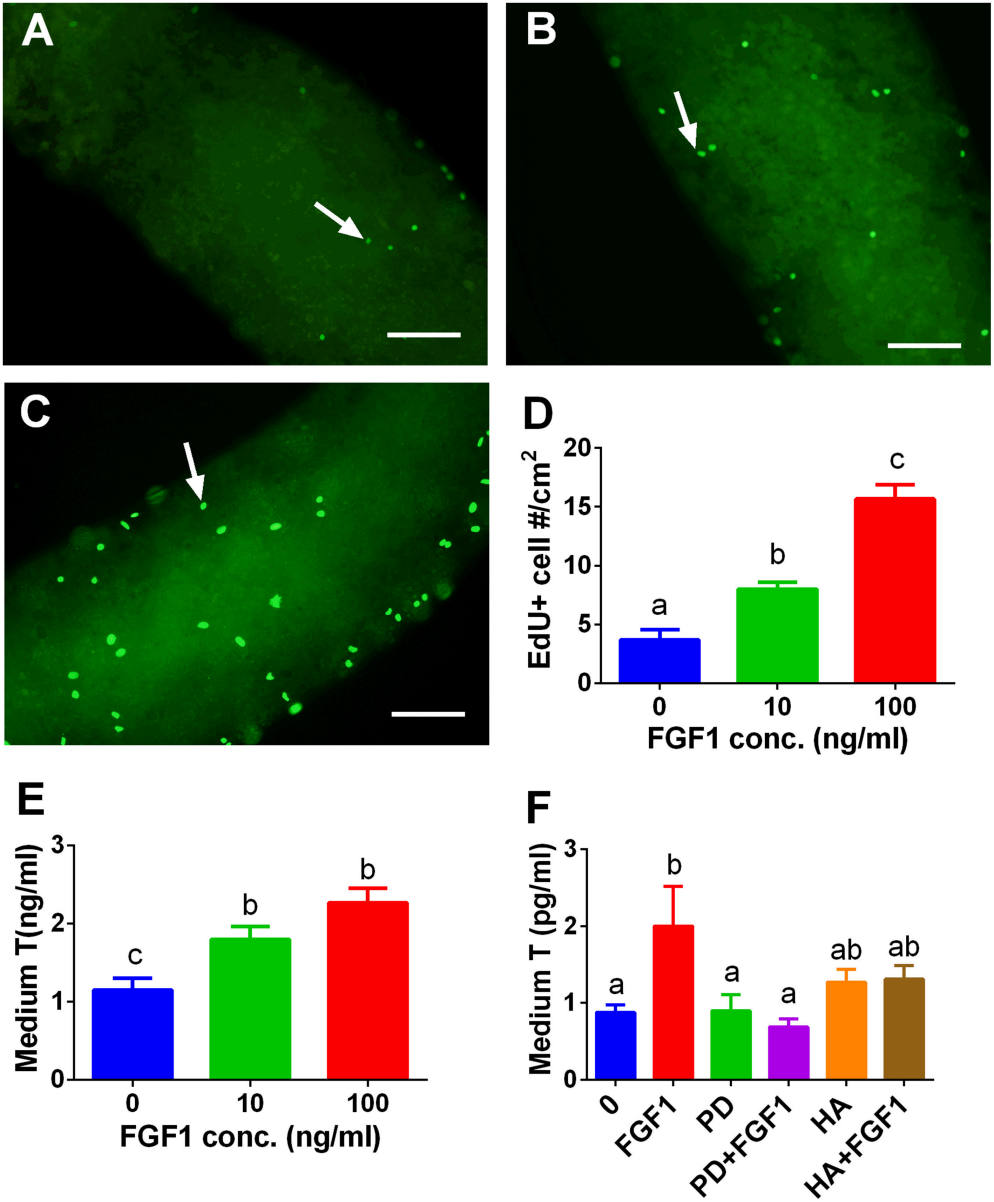
EdU incorporation into stem Leydig cells and indirect measurement of medium testosterone (T) levels after FGF1 treatment *in vitro*. Immunofluorescent staining of EdU (green color in the nucleus) of stem Leydig cells on the surface of the seminiferous tubules treated with 0 **(A)**, 10 **(B)**, and 100 ng/ml FGF1 **(C)** for 7 days; **(D)**: quantitative data of percentage of EdU-positive cells per cm^2^, Mean ± SEM, *n* = 3. White arrow designates EdU-positive stem Leydig cells. Bar = 50 μm. **(E,F)**, medium testosterone (T) levels after the seminiferous tubules were treated initially by FGF1 and/or 1 μM PD173074 (PD) and/or 1 μg/ml heparin (HA) for 7 days and then the tubules were switched in LCDM for additional 2 weeks. Mean ± SEM, *n* = 6. Identical letters designate no significant differences at *P* < 0.05 between two groups.

We tested whether FGF1 influence SLC proliferation. EdU incorporation into the SLCs on the surface of the STs was used. Indeed, FGF1 increased the EdU incorporation into SLCs after 10 and 100 ng/ml FGF1 (Figure 9D). We also judged the effects of FGF1 on SLC proliferation by increasing the number of SLCs during the first week of FGF1 treatment and subsequently inducing them to differentiate into ALCs in LCDM for 2 weeks. Indeed, FGF1 increased the medium testosterone levels after the initial 1-week treatment (Figure 9E). When 100 ng/ml FGF1 was incubated with or without 1 μM PD173074 or 1μg/ml heparin, PD173074 or heparin alone did not affect T levels. However, they reversed FGF1-mediated responses (Figure 9F), indicating that FGF1 acts via FGFR1 and requires HSPG as the partner. We further detected mRNA levels of *Lhcgr, Scarb1, Star, Cyp11a1, Hsd3b1, Cyp17a1, Hsd17b3, Srd5a1*, and *Hsd11b1*. We observed that FGF1 at 100 ng/ml increased *Cyp11a1, Hsd3b1*, and *Cyp17a1* mRNA levels (Figure S3).

## Discussion

FGFs have been demonstrated to control sex determination (31). Herein, we identified that SLCs express higher level of *Fgfr1* and FGF1 is able to increase the proliferation of SLCs, thus populating Leydig cell number during its earlier stage of regeneration in an EDS-treated model.

FGF1 has been found to be present in many cell types in rat testis, including Leydig and peritubular cells, and four FGFRs (FGFR1, FGFR2, FGFR3, and FGFR4) have been found to exist in the Leydig cell lineage (15). This suggests that FGF1 can act through all these receptors via autocrine and paracrine (15). Here, we reanalyzed the microarray data from the purified cells (stem, progenitor, immature, and adult Leydig cells) and found that *Fgfr1* and *Fgfr3* are present in the ALC lineage, including the SLCs (Figure S1), and *Fgfr1* level is much higher than that of *Fgfr3*, indicating that SLCs are also the target of FGF1. While other *Fgfrs* were not detectable in this array, we suggest that SLCs contains a major FGFR1 and a minor FGFR3.

In the EDS-treated ALC regeneration model, there is a dramatic increase of cell number through the initial proliferation of SLCs (32). We treated EDS-administered testis with FGF1 and found that FGF1 significantly increased Leydig cell proliferation as shown by the increase of PCNA-labeling Leydig cell percentage (Figure 5) and Leydig cell number. We further demonstrated that FGF1 indeed increased SLC proliferation as shown by the increase of EdU incorporation and increased medium testosterone levels after the treatment of FGF1 during the SLC proliferative phase (Figure 9). FGF1 stimulated SLC proliferation via acting on FGFR1, since its antagonist PD173074 can reverse FGF1 action (Figure 9). Heparin, which blocks HSPG activity, can also reverse FGF1 action (Figure 9). This indicates that FGF1 binds FGFR1 requiring HSPG activity. Indeed, a previous study showed that FGF alone inhibited LH-stimulated testosterone production by rat adult Leydig cells in the presence of HSPG (13). Therefore, we suggest that FGF1 stimulates SLC proliferation via binding to FGFR1 which is coupling with HSPG to take an action.

Interestingly, FGF1 remarkably down-regulated Leydig cell specific gene expression in the Leydig cell *per se*. Thus, in the EDS-regenerated model, the serum testosterone levels rely on the balance of Leydig cell number and Leydig cell steroidogenesis *per se*. Indeed, FGF1 significantly up-regulated *Scarb1* and *Hsd17b3* expression without Leydig cell number adjustment and increased serum testosterone levels at 100 ng/testis (Figure 1). Therefore, the increase of serum testosterone is mainly from the increased Leydig cell number but not the capacity of each Leydig cell.

Apparently, FGF1 blocked the differentiation of SLCs into the ALC lineage *in vitro*. To support this, we found that FGF1 lowered testosterone amount in the medium and down-regulated Leydig cell-specific gene expression. FGF1 exerted its stimulation of SLC proliferation and blockade of SLC differentiation via FGFR1 because the FGFR1 antagonist PD173074 significantly reversed FGF1 action on both proliferation and differentiation.

In addition to its effects on SLC function, FGF1 might also control the Leydig cell function. FGF-1 has been demonstrated to stimulate 5α-androstane-3α,17β-diol production by primary Leydig cells isolated from immature rats under the basal condition (13). However, FGF1 blocked LH-stimulated androgen production in rat Leydig cells, by lowering LHCGR number and HSD3B1 and SRD5A1 activities (16). LH also inhibited LH-stimulated testosterone production by rat ALCs (13). LH is an important factor for the pubertal development and regeneration of Leydig cells since knockout of LHCGR blocked the late stage of development of Leydig cells as shown by the only presence of progenitor cells in these knockout mice (33, 34). Thus, the suppression of LH-stimulated action by FGF1 could block the differentiation of progenitor into immature Leydig cells. Indeed, FGF1 *in vitro* significantly lowered medium testosterone levels and down-regulated Leydig cell-specific gene expression, suggesting that FGF1 blocks both the differentiation of SLCs and progenitor cells.

FGF1 binds to FGFR1 and exerts its effects on SLC development. FGF1/FGFR1 pathway includes direct recruitment of proteins that bind to tyrosine autophosphorylation sites and/or indirect recruitment of docking proteins that are phosphorylated by tyrosine kinases after FGFR1 activation. FGF1/FGFR transduction activates several pathways, including the phosphatidylinositol 3-kinase (PI3K)/AKT pathways. FGF1-FGFR1 signaling may be mediated by down-stream AKT1 and AKT2 as well as ERK pathways (35). AKT is a critical regulator of cell development. Three isoforms of AKT in mammals were found, AKT1-AKT3. AKT1 is the major isoform in numerous mammalian tissues and it controls each organ growth; AKT2 is present in tissues to regulate glucose metabolism; and AKT3 is primarily expressed in the brain to regulate brain function (36). AKT1 knock in mice causes the testis abnormality (37) while AKT2-AKT3 double knockout in mice does not induce any abnormality of the testis, (38) indicating that AKT1 is a major downstream signaling to regulate testis function. AKT1 is mainly controlled by insulin-like growth factor 1 via PI3K (39) and insulin-like growth factor 1 knockout in mice blocks Leydig cell proliferation (40, 41). Indeed, the stimulation of phosphorylation of AKT1 by FGF1 may contribute to its mediated elevation of SLC proliferation.

MEK-ERK1/2 pathway is a critical signaling pathway that mediates many signals from the surface receptors such as epidermal growth factors and FGF. MEK phosphorylates ERK1/2, activating the down-stream cascades. It has been reported that a Leydig cell conditional double knockout of MEK1/2, the upstream kinases of ERK1/2, induces Leydig cell hypoplasia and the decreased androgen production as well as the down-regulation of steroidogenesis-related genes, including *Cyp17a1*, in mice (42). Here, we did not observe the change of ERK1/2 phosphorylation after FGF1 treatment, indicating that this pathway may not be involved in FGF1-mediated regulation.

The dose (100 ng/testis) for testicular injection may be higher. However, for *in vivo s*tudy, due to the circulation, such concentration may be needed to produce significant effects. Perry et al. uses 10 μg ICV to treat diabetic rats (43). The dose we used is far lower than that in Perry's study.

**Figure 10:**
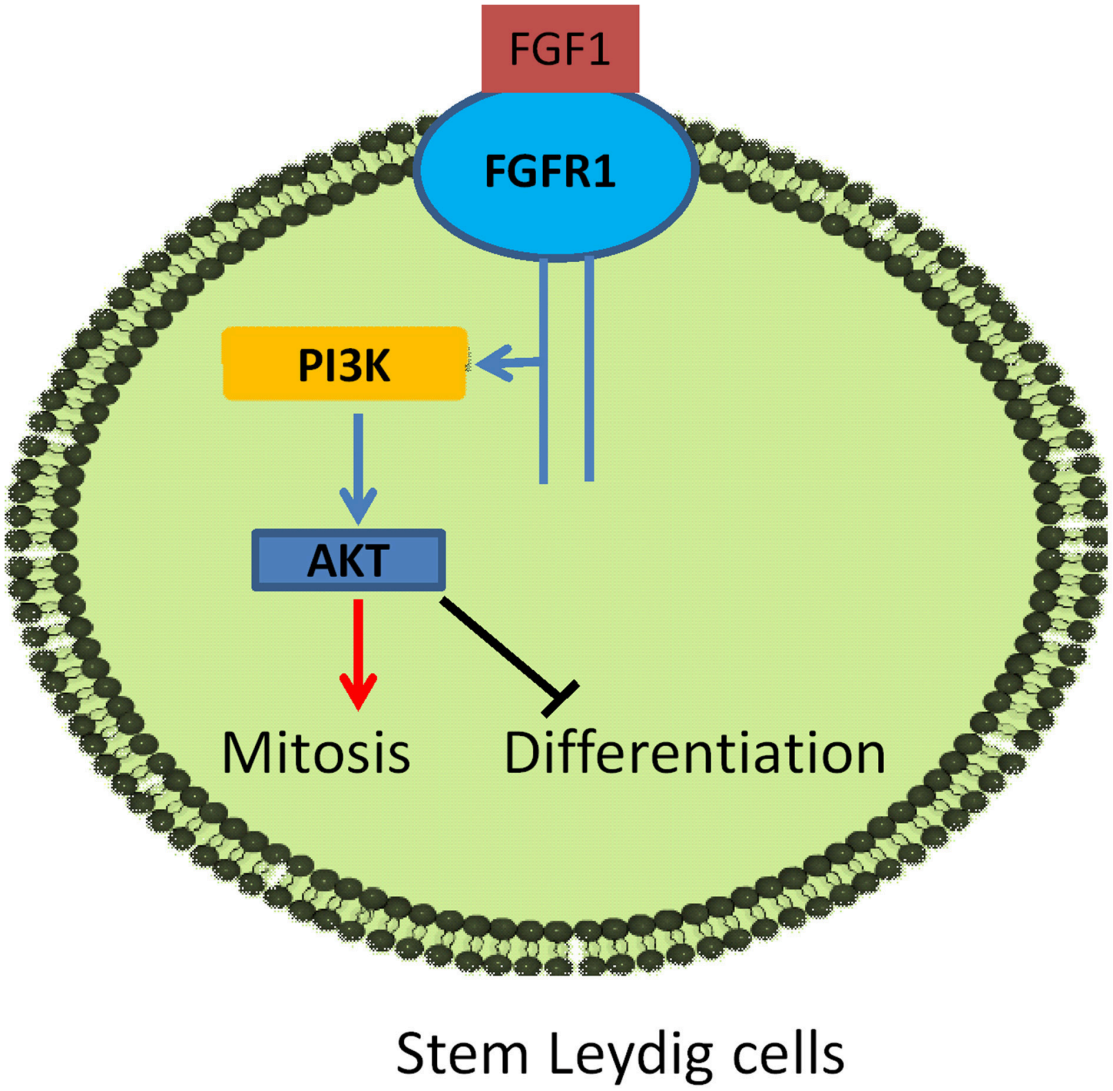
Illustration of FGF1 signaling in Leydig cell development. FGF1 binds to FGFR1, activating PI3K/AKT pathway, leading to the increase of stem Leydig cell mitosis and the suppression of its differentiation.

In conclusion, FGF1 binds to stem/progenitor Leydig cell FGFR1 which interacts with HSPG to stimulate stem/progenitor Leydig cell proliferation and to block their differentiation (Figure 10), possibly via AKT1 phosphorylation pathway.

## Author Contributions

XinL and R-SG conceptualized the study design and analyze the data. LC, XiaL, YW, TS, HL, LX, LL, XC, LM, YC, and YL performed the experiments and collected the data. R-SG wrote the manuscript.

### Conflict of Interest Statement

The authors declare that the research was conducted in the absence of any commercial or financial relationships that could be construed as a potential conflict of interest.

## Supplementary Material

Figure S1 Expression levels of Fgfr1 and Fgfr3 in the Leydig cell lineage. The microarray of transcriptome data of stem (SLC), progenitor (PLC), immature (ILC), and adult (ALC) Leydig cells was reanalyzed. Mean ± SEM, *n* = 4. Asterisks (^*^, ^**^, and ^***^) designate significant differences between *Fgfr1* and *Fgfr3* at *P* < 0.05, 0.01, and 0.001, respectively, at each cell type.

Figure S2 FGF1 affects the expression levels of Leydig cell-specific genes adjusted by Leydig cell number *in vivo*. The mRNA levels of *Lhcgr, Scarb1, Star, Cyp11a1, Hsd3b1, Cyp17a1, Hsd17b3, Srd5a1*, and *Hsd11b1* were analyzed by qPCR in the testes from rats treated with 0, 10, and 100 ng/testis FGF1 on post-EDS day 14 for 14 days. The mRNA levels were adjusted by CYP11A1-positive cells. Mean ± SEM, *n* = 6, Asterisks (^*^, ^**^, ^***^) designate significant difference at *P* < 0.05, 0.01, and 0.001, respectively, when compared to the control (0 ng/testis FGF1).

Figure S3 FGF1 affects the expression levels of Leydig cell-specific genes after the initial FGF1 treatment *in vitro*. The mRNA levels of *Lhcgr, Scarb1, Star, Cyp11a1, Hsd3b1, Cyp17a1, Hsd17b3, Srd5a1*, and *Hsd11b1* were analyzed by qPCR in the seminiferous tubules from rats treated with 0, 10, and 100 ng/ml FGF1 for 7 days followed by the culture in LCDM for 14 days. The mRNA levels were adjusted by *Rps16*. Mean ± SEM, *n* = 6, Asterisks (^*^, ^**^, ^***^) designate significant difference at *P* < 0.05, 0.01, and 0.001, respectively, when compared to the control (0 ng/ml FGF1).

Supplementary Table S1 Primer information.

Supplementary Table S2 Antibodies.

Supplementary Table S3 Body weight and testis weight after FGF1 treatment for 14 days.

## References

[1] Molenaar R de Rooij DG Rommerts FF Reuvers PJ van der Molen HJ. Specific destruction of Leydig cells in mature rats after *in vivo* administration of ethane dimethyl sulfonate. Biol Reprod. (1985) 33:1213–22. 10.1095/biolreprod33.5.1213 3000465

[2] Bartlett JM Kerr JB Sharpe RM. The effect of selective destruction and regeneration of rat Leydig cells on the intratesticular distribution of testosterone and morphology of the seminiferous epithelium. J Androl. (1986) 7:240–53. 10.1002/j.1939-4640.1986.tb00924.x 3745011

[3] Ge RS Dong Q Sottas CM Papadopoulos V Zirkin BR Hardy MP. In search of rat stem Leydig cells: identification, isolation, and lineage-specific development. Proc Natl Acad Sci USA. (2006) 103:2719–24. 10.1073/pnas.0507692103 16467141 PMC1413776

[4] Guo J Zhou B Chen H Su Z Wang G Wang CQF. Comparison of cell types in the rat leydig cell lineage after ethane dimethanesulfonate treatment. Reproduction. (2013) 145:371–80. 10.1530/REP-12-0465 23587774

[5] Zhang YF Yuan KM Liang Y Chu YH Lian QQ Ge YF. Alterations of gene profiles in Leydig-cell-regenerating adult rat testis after ethane dimethane sulfonate-treatment. Asian J Androl. (2015) 17:253–60. 10.4103/1008-682X.136447 25337835 PMC4405920

[6] Stanley E Lin CY Jin S Liu J Sottas CM Ge R. Identification, proliferation, and differentiation of adult Leydig stem cells. Endocrinology. (2012) 153:5002–10. 10.1210/en.2012-1417 22865373 PMC3512003

[7] Li X Wang Z Jiang Z Guo J Zhang Y Li C. Regulation of seminiferous tubule-associated stem Leydig cells in adult rat testes. Proc Natl Acad Sci USA. (2016) 113:2666–71. 10.1073/pnas.1519395113 26929346 PMC4790979

[8] Gaytan F Bellido C Aguilar E van Rooijen N. Requirement for testicular macrophages in Leydig cell proliferation and differentiation during prepubertal development in rats. J Reprod Fertil. (1994) 102:393–9. 10.1530/jrf.0.1020393 7861393

[9] Beenken A Mohammadi M. The FGF family: biology, pathophysiology and therapy. Nat Rev Drug Discov. (2009) 8:235–53. 10.1038/nrd2792 19247306 PMC3684054

[10] Nies VJ Sancar G Liu W van Zutphen T Struik D Yu RT. Fibroblast growth factor signaling in metabolic regulation. Front Endocrinol. (2015) 6:193. 10.3389/fendo.2015.00193 26834701 PMC4718082

[11] Eswarakumar VP Lax I Schlessinger J. Cellular signaling by fibroblast growth factor receptors. Cytokine Growth Factor Rev. (2005) 16:139–49. 10.1016/j.cytogfr.2005.01.001 15863030

[12] Zhang X Ibrahimi OA Olsen SK Umemori H Mohammadi M Ornitz DM. Receptor specificity of the fibroblast growth factor family. The complete mammalian FGF family. J Biol Chem. (2006) 281:15694–700. 10.1074/jbc.M601252200 16597617 PMC2080618

[13] Laslett AL McFarlane JR Risbridger GP. Developmental response by Leydig cells to acidic and basic fibroblast growth factor. J Steroid Biochem Mol Biol. (1997) 60:171–9. 10.1016/S0960-0760(96)00180-X 9191974

[14] Harmer NJ Ilag LL Mulloy B Pellegrini L Robinson CV Blundell TL. Towards a resolution of the stoichiometry of the fibroblast growth factor (FGF)-FGF receptor-heparin complex. J Mol Biol. (2004) 339:821–34. 10.1016/j.jmb.2004.04.031 15165853

[15] Cancilla B Davies A Ford-Perriss M Risbridger GP. Discrete cell- and stage-specific localisation of fibroblast growth factors and receptor expression during testis development. J Endocrinol. (2000) 164:149–59. 10.1677/joe.0.1640149 10657850

[16] Murono EP Washburn AL Goforth DP Wu N. Effects of acidic fibroblast growth factor on 5-ene-3 beta-hydroxysteroid dehydrogenase-isomerase and 5 alpha-reductase activities and [125I]human chorionic gonadotrophin binding in cultured immature Leydig cells. J Steroid Biochem Mol Biol. (1993) 45:477–83. 10.1016/0960-0760(93)90162-P 7686040

[17] Stanley EL Johnston DS Fan J Papadopoulos V Chen H Ge RS. Stem Leydig cell differentiation: gene expression during development of the adult rat population of Leydig cells. Biol Reprod. (2011) 85:1161–6 10.1095/biolreprod.111.091850 21832170 PMC3223250

[18] Wu X Guo X Wang H Zhou S Li L Chen X. A brief exposure to cadmium impairs Leydig cell regeneration in the adult rat testis. Sci Rep. (2017) 7:6337. 10.1038/s41598-017-06870-0 28740105 PMC5524795

[19] Zhang L Wang H Yang Y Liu H Zhang Q Xiang Q. NGF induces adult stem Leydig cells to proliferate and differentiate during Leydig cell regeneration. Biochem Biophys Res Commun. (2013) 436:300–5 10.1016/j.bbrc.2013.05.098 23743199

[20] Chen H Stanley E Jin S Zirkin BR. Stem Leydig cells: from fetal to aged animals. Birth Defects Res C Embryo Today. (2010) 90:272–83. 10.1002/bdrc.20192 21181888 PMC4103425

[21] Liu J Wang Y Fang Y Ni C Ma L Zheng W. Gestational exposure to ziram disrupts rat fetal Leydig cell development. Chemosphere. (2018) 203:393–401. 10.1016/j.chemosphere.2018.03.142 29627606

[22] Mendis-Handagama SM Keeney DS Hardy MP Ewing LL. Application of the disector method to enumerate cells in the testis. Ann NY Acad Sci. (1989) 564:86–98. 10.1111/j.1749-6632.1989.tb25890.x 2774419

[23] Kerr JB Donachie K Rommerts FF. Selective destruction and regeneration of rat Leydig cells *in vivo*. A new method for the study of seminiferous tubular-interstitial tissue interaction. Cell Tissue Res. (1985) 242:145–56. 10.1007/BF00225571 4042133

[24] Vihko KK Suominen JJ Parvinen M. Cellular regulation of plasminogen activator secretion during spermatogenesis. Biol Reprod. (1984) 31:383–9. 10.1095/biolreprod31.2.383 6541063

[25] Song T Wang Y Li H Chen L Liu J Chen X. Parathyroid hormone-related protein promotes rat stem leydig cell differentiation. Front Physiol. (2017) 8:911. 10.3389/fphys.2017.00911 29180966 PMC5693895

[26] Rommerts FF Teerds KJ Hoogerbrugge JW. *In vitro* effects of ethylene-dimethane sulfonate (EDS) on Leydig cells: inhibition of steroid production and cytotoxic effects are dependent on species and age of rat. Mol Cell Endocrinol. (1988) 55:87–94. 10.1016/0303-7207(88)90094-9 2834244

[27] Teerds KJ De Rooij DG Rommerts FF Wensing CJ The regulation of the proliferation and differentiation of rat Leydig cell precursor cells after EDS administration or daily HCG treatment. J Androl. (1988) 9:343–51. 10.1002/j.1939-4640.1988.tb01061.x 2853150

[28] Manna PR Chandrala SP King SR Jo Y Counis R Huhtaniemi IT. Molecular mechanisms of insulin-like growth factor-I mediated regulation of the steroidogenic acute regulatory protein in mouse leydig cells. Mol Endocrinol. (2006) 20:362–78. 10.1210/me.2004-0526 16166197

[29] Manna PR Jo Y Stocco DM. Regulation of Leydig cell steroidogenesis by extracellular signal-regulated kinase 1/2: role of protein kinase A and protein kinase C signaling. J Endocrinol. (2007) 193:53–63. 10.1677/JOE-06-0201 17400803

[30] Shiraishi K Ascoli M. Lutropin/choriogonadotropin stimulate the proliferation of primary cultures of rat Leydig cells through a pathway that involves activation of the extracellularly regulated kinase 1/2 cascade. Endocrinology. (2007) 148:3214–25. 10.1210/en.2007-0160 17412805 PMC2085235

[31] Chen H Wang Y Ge R Zirkin BR Leydig cell stem cells: Identification, proliferation and differentiation. Mol Cell Endocrinol. (2016). 10.1016/j.mce.2016.10.010 PMC5346484 27743991

[32] Teerds KJ De Rooij DG Rommerts FF van den Hurk R Wensing CJ. Stimulation of the proliferation and differentiation of Leydig cell precursors after the destruction of existing Leydig cells with ethane dimethyl sulphonate (EDS) can take place in the absence of LH. J Androl. (1989) 10:472–7. 10.1002/j.1939-4640.1989.tb00143.x 2559907

[33] Lei ZM Mishra S Zou W Xu B Foltz M Li X. Targeted disruption of luteinizing hormone/human chorionic gonadotropin receptor gene. Mol Endocrinol. (2001) 15:184–200. 10.1210/mend.15.1.0586 11145749

[34] Zhang FP Poutanen M Wilbertz J Huhtaniemi I Normal prenatal but arrested postnatal sexual development of luteinizing hormone receptor knockout (LHRKO) mice. Mol Endocrinol. (2001) 15:172 10.1210/mend.15.1.0582 11145748

[35] Ornitz DM Itoh N. The fibroblast growth factor signaling pathway. Wiley Interdiscip Rev Dev Biol. (2015) 4:215–66. 10.1002/wdev.176 25772309 PMC4393358

[36] Hay N. Akt isoforms and glucose homeostasis - the leptin connection. Trends Endocrinol Metab. (2011) 22:66–73. 10.1016/j.tem.2010.09.003 20947368 PMC3427792

[37] Chen WS Xu PZ Gottlob K Chen ML Sokol K Shiyanova T. Growth retardation and increased apoptosis in mice with homozygous disruption of the Akt1 gene. Genes Dev. (2001) 15:2203–8. 10.1101/gad.913901 11544177 PMC312770

[38] Dummler B Tschopp O Hynx D Yang ZZ Dirnhofer S Hemmings BA. Life with a single isoform of Akt: mice lacking Akt2 and Akt3 are viable but display impaired glucose homeostasis and growth deficiencies. Mol Cell Biol. (2006) 26:8042–51. 10.1128/MCB.00722-06 16923958 PMC1636753

[39] Tai P Shiraishi K Ascoli M. Activation of the lutropin/choriogonadotropin receptor inhibits apoptosis of immature Leydig cells in primary culture. Endocrinology. (2009) 150:3766–73 10.1210/en.2009-0207 19406941 PMC2717876

[40] Baker J Hardy MP Zhou J Bondy C Lupu F Bellve AR. Effects of an Igf1 gene null mutation on mouse reproduction. Mol Endocrinol. (1996) 10:903–18. 8813730 10.1210/mend.10.7.8813730

[41] Hu GX Lin H Chen GR Chen BB Lian QQ Hardy DO. Deletion of the Igf1 gene: suppressive effects on adult Leydig cell development. J Androl. (2010) 31:379–87. 10.2164/jandrol.109.008680 20203337 PMC4103413

[42] Matzkin ME Yamashita S Ascoli M. The ERK1/2 pathway regulates testosterone synthesis by coordinately regulating the expression of steroidogenic genes in Leydig cells. Mol Cell Endocrinol. (2013) 370:130–7. 10.1016/j.mce.2013.02.017 23480967 PMC3631444

[43] Perry RJ Lee S Ma L Zhang D Schlessinger J Shulman G. FGF1 and FGF19 reverse diabetes by suppression of the hypothalamic-pituitary-adrenal axis. Nat Commun. (2015) 6:6980. 10.1038/ncomms7980 25916467 PMC4413509

