# Supplementary figures and images for: Fibroblast Growth Factor 1 Promotes Rat Stem Leydig Cell Development
Source: Front Endocrinol (Lausanne). 2019 Mar 8;10:118. doi: 10.3389/fendo.2019.00118 (PMC6418010; doi:10.3389/fendo.2019.00118)

**FigureS1**

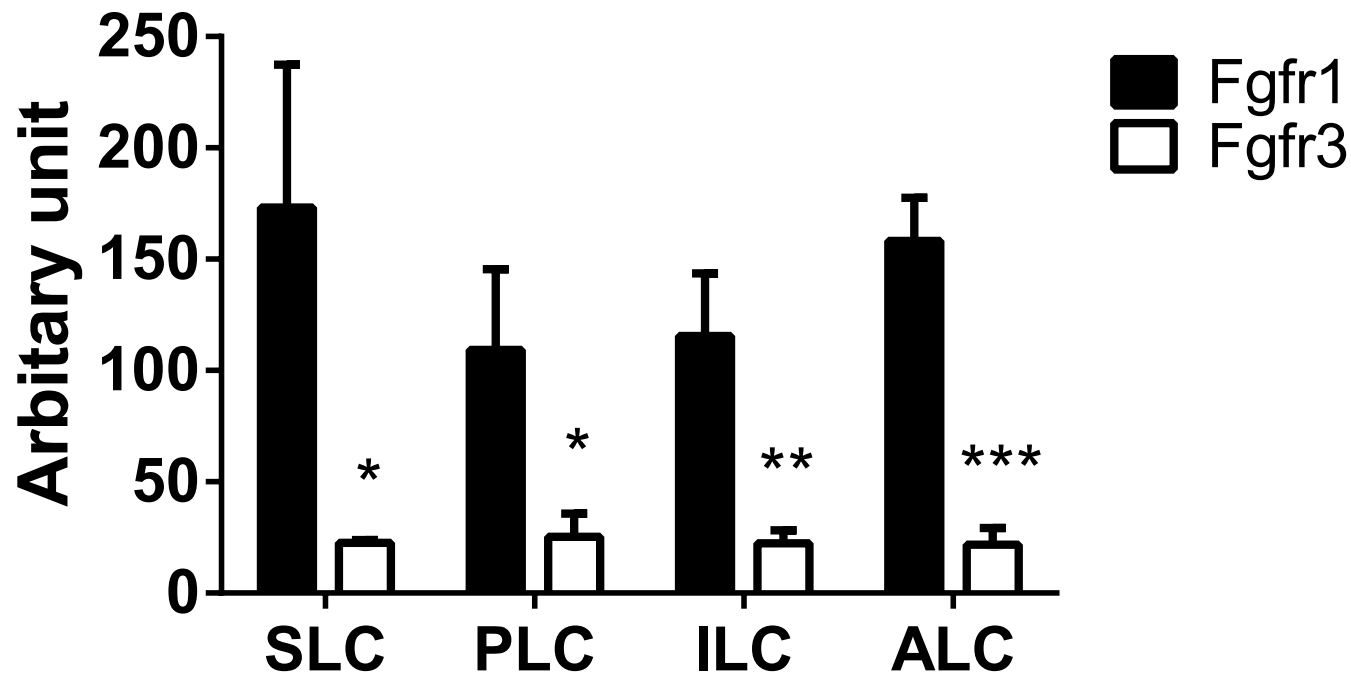

Supplement: Figure S1 — Expression levels of Fgfr1 and Fgfr3 in the Leydig cell lineage. The microarray of transcriptome data of stem (SLC), progenitor (PLC), immature (ILC), and adult (ALC) Leydig cells was reanalyzed. Mean ± SEM, n = 4. Asterisks (*, **, and ***) designate significant differences between Fgfr1 and Fgfr3 at P < 0.05, 0.01, and 0.001, respectively, at each cell type. [file Image_1.pdf]

**FigureS2**

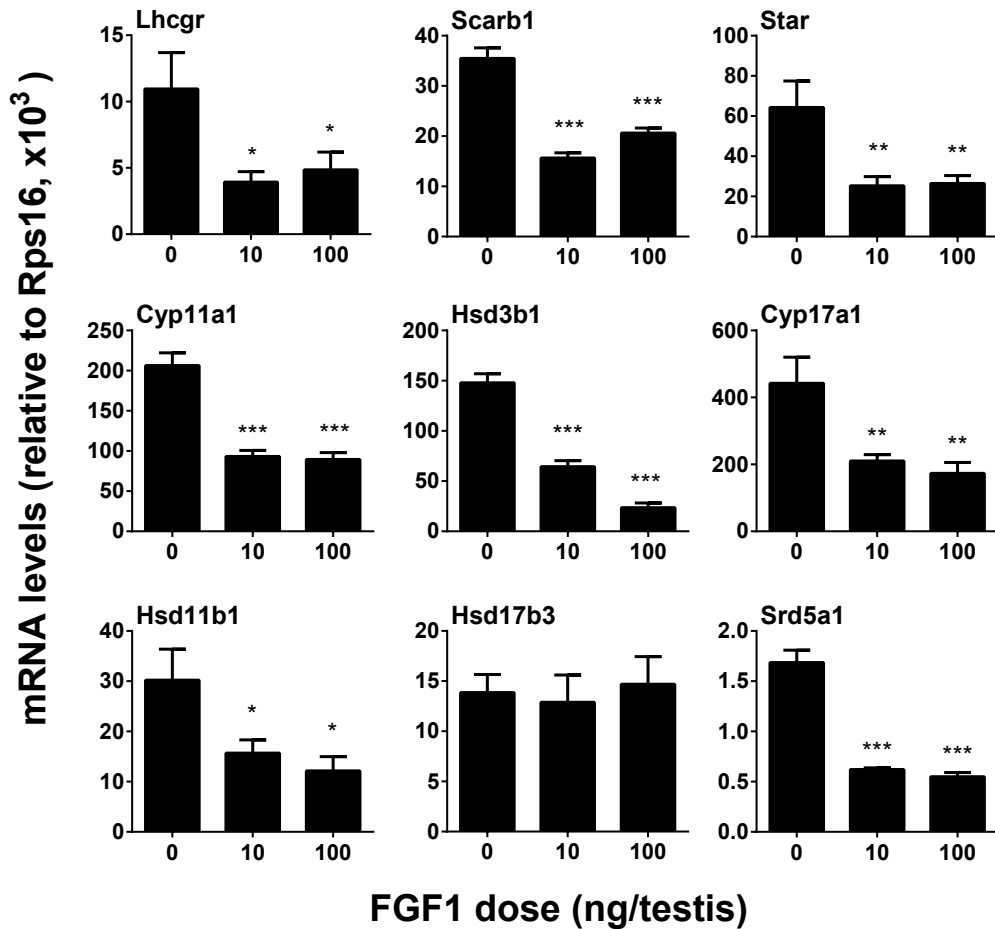

Supplement: Figure S2 — FGF1 affects the expression levels of Leydig cell-specific genes adjusted by Leydig cell number in vivo. The mRNA levels of Lhcgr, Scarb1, Star, Cyp11a1, Hsd3b1, Cyp17a1, Hsd17b3, Srd5a1, and Hsd11b1 were analyzed by qPCR in the testes from rats treated with 0, 10, and 100 ng/testis FGF1 on post-EDS day 14 for 14 days. The mRNA levels were adjusted by CYP11A1-positive cells. Mean ± SEM, n = 6, Asterisks (*, **, ***) designate significant difference at P < 0.05, 0.01, and 0.001, respectively, when compared to the control (0 ng/testis FGF1). [file Image_2.pdf]

**FigureS3**

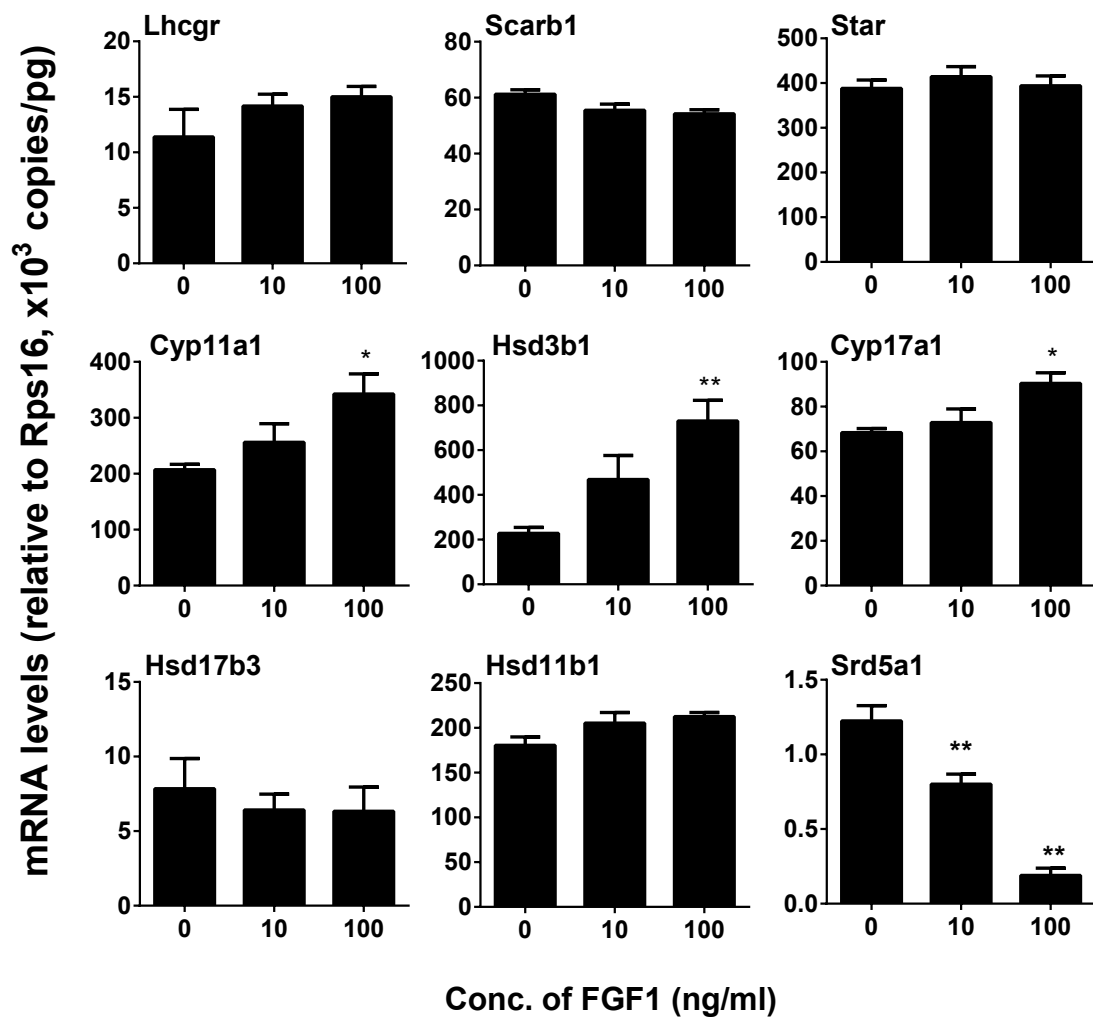

Supplement: Figure S3 — FGF1 affects the expression levels of Leydig cell-specific genes after the initial FGF1 treatment in vitro. The mRNA levels of Lhcgr, Scarb1, Star, Cyp11a1, Hsd3b1, Cyp17a1, Hsd17b3, Srd5a1, and Hsd11b1 were analyzed by qPCR in the seminiferous tubules from rats treated with 0, 10, and 100 ng/ml FGF1 for 7 days followed by the culture in LCDM for 14 days. The mRNA levels were adjusted by Rps16. Mean ± SEM, n = 6, Asterisks (*, **, ***) designate significant difference at P < 0.05, 0.01, and 0.001, respectively, when compared to the control (0 ng/ml FGF1). [file Image_3.pdf]
