# Supplementary material for: Fibroblast Growth Factor 1 Promotes Rat Stem Leydig Cell Development
Source: Front Endocrinol (Lausanne). 2019 Mar 8;10:118. doi: 10.3389/fendo.2019.00118 (PMC6418010; doi:10.3389/fendo.2019.00118)
Supplement: Supplementary Table S1 — Primer information. [file Table_1.doc]

**Supplementary Table S1. Primer information**

| **Primer**  **symbol** | **Primer direction** | **Sequences (5’to 3’)** | **Product length (bp)** | **Accession** |
| --- | --- | --- | --- | --- |
| Lhcgr | Forward | CTGCGCTGTCCTGGCC | 103 | NM_012978 |
|  | Reverse | CGACCTCATTAAGTCCCCTGAA |  |  |
| Scarb1 | Forward | ATGGTACTGCCGGGCAGAT | 117 | NM_031541 |
|  | Reverse | CGAACACCCTTGATTCCTGGTA |  |  |
| Star | Forward | CCCAAATGTCAAGGAAATCA | 187 | NM_031558 |
|  | Reverse | AGGCATCTCCCCAAAGTG |  |  |
| Cyp11a1 | Forward | AAGTATCCGTGATGTGGG | 127 | NM_017286 |
|  | Reverse | TCATACAGTGTCGCCTTTTCT |  |  |
| Hsd3b1 | Forward | CCCTGCTCTACTGGCTTGC | 189 | NM_001007719 |
|  | Reverse | TCTGCTTGGCTTCCTCCC |  |  |
| Cyp17a1 | Forward | TGGCTTTCCTGGTGCACAATC | 90 | NM_012753 |
|  | Reverse | TGAAAGTTGGTGTTCGGCTGAAG |  |  |
| Hsd17b3 | Forward | TGAAAGTTGGTGTTCGGCTGAAG | 202 | NM_054007 |
|  | Reverse | TGAAAGTTGGTGTTCGGCTGAAG |  |  |
| Srd5a1 | Forward | TCACCAGAGCGAAGCAGC | 92 | NM_017070 |
|  | Reverse | CTAAAGCACAAATGGAATCAGTAT |  |  |
| Hsd11b1 | Forward | TCTTCTTGGCCTACTACTAC | 91 | J05107 |
|  | Reverse | TTGCTGGCCCCTGTGACAAT |  |  |
| Rps16 | Forward | AAGTCTTCGGACGCAAGAAA | 148 | XM_341815 |
|  | Reverse | TTGCCCAGAAGCAGAACAG |  |  |
