# Supplementary material for: Fibroblast Growth Factor 1 Promotes Rat Stem Leydig Cell Development
Source: Front Endocrinol (Lausanne). 2019 Mar 8;10:118. doi: 10.3389/fendo.2019.00118 (PMC6418010; doi:10.3389/fendo.2019.00118)
Supplement: Supplementary Table S2 — Antibodies. [file Table_2.DOC]

**Supplementary table S2. Antibodies**

| **Antibody** | **Species** | **Vendor (City, State)** | | **Dilution** | | | |
| --- | --- | --- | --- | --- | --- | --- | --- |
|  |  |  |  | **WB** | | **HS** | |
| Actin (ACTB) | Mouse | Beyotime (Shanghai, China) | | 1:1000 | | ND | |
| LHCGR  SCARB1 | Rabbit Rabbit | Multi Sciences (Hangzhou, China)  Multi Sciences (Hangzhou, China) | | 1:1000  1:1000 | | ND  ND | |
| CYP11A1 | Rabbit | Cell Signaling Technology (Danvers, MA) | ND | | 1:200 | |  |
| HSD3B1 | Rabbit | Multi Sciences (Hangzhou, China) | | 1:500 | | ND | |
| CYP17A1  HSD17B3 | Rabbit  Rabbit | Abcam (San Francisco, CA)  Abcam (San Francisco, CA) | | 1:1000  1:1000 | | ND  ND | |
| HSD11B1  pAKT1  AKT1  pAKT2  AKT2  pERK  ERK  PCNA  α- SMA | Rabbit  Rabbit  Rabbit  Rabbit  Rabbit  Mouse  Mouse  Mouse  Mouse | Abcam (San Francisco, CA)  Abcam (San Francisco, CA)  Abcam (San Francisco, CA)  Abcam (San Francisco, CA)  Abcam (San Francisco, CA)  Abcam (San Francisco, CA)  Abcam (San Francisco, CA)  Abcam (San Francisco, CA)  Sigma-Aldrich (St. Louis, MO) | | 1:1000  1:2000  1:1000  1:1000  1:1000  1:1000  1:1000  ND | | 1:200  ND  ND  ND  ND  ND  ND  1:50  1:200 | |

ND = not detected.
