# Supplementary material for: Fibroblast Growth Factor 1 Promotes Rat Stem Leydig Cell Development
Source: Front Endocrinol (Lausanne). 2019 Mar 8;10:118. doi: 10.3389/fendo.2019.00118 (PMC6418010; doi:10.3389/fendo.2019.00118)
Supplement: Supplementary Table S3 — Body weight and testis weight after FGF1 treatment for 14 days. [file Table_3.DOC]

**Supplementary Table S3. Body weight and testis weight after FGF1 treatment for 14 days**

| Parameters | | | | **FGF1 dosage (ng/testis)** | | | |
| --- | --- | --- | --- | --- | --- | --- | --- |
|  |  | | **0** | | **100** | **1000** | |
| **Body weight (g)** | | 396.3±17.16 | | | 384.9±15.8 | | 397.9±14.08 |
| **Testis weight (g)** | | 1.564±0.05341 | | | 1.541±0.08605 | | 1.491±0.05497 |
| **Testis weight/ Body weight (×1000)** | | 3.94±0.201 | | | 3.944±0.2154 | | 3.746±0.1456 |

Mean ± SEM, n=6.
